# DOT1L inhibitor improves early development of porcine somatic cell nuclear transfer embryos

**DOI:** 10.1371/journal.pone.0179436

**Published:** 2017-06-20

**Authors:** Jia Tao, Yu Zhang, Xiaoyuan Zuo, Renyun Hong, Hui Li, Xing Liu, Weiping Huang, Zubing Cao, Yunhai Zhang

**Affiliations:** Anhui Provincial Key Laboratory of Local Livestock and Poultry Genetic Resource Conservation and Bio-Breeding, College of Animal Science and Technology, Anhui Agricultural University, Hefei, Anhui, China; University of Florida, UNITED STATES

## Abstract

Incomplete epigenetic reprogramming of the genome of donor cells causes poor early and full-term developmental efficiency of somatic cell nuclear transfer (SCNT) embryos. Previous research indicate that inhibition of the histone H3 K79 methyltransferase DOT1L, using a selective pharmacological inhibitor EPZ004777 (EPZ), significantly improved reprogramming efficiency during the generation of mouse induced pluripotent stem cells. However, the roles of DOT1L in porcine nuclear transfer-mediated cellular reprogramming are not yet known. Here we showed that DOT1L inhibition via 0.5 nM EPZ treatment for 12 or 24 h significantly enhanced the blastocyst rate of SCNT embryos and dramatically reduced the level of H3K79me2 during SCNT 1-cell embryonic development. Additionally, H3K79me2 level in the EPZ-treated SCNT embryos was similar to that in *in vitro* fertilized embryos, suggesting that DOT1L-mediated H3K79me2 is a reprogramming barrier to early development of porcine SCNT embryos. qRT-PCR analysis further demonstrated that DOT1L inactivation did not change the expression levels of *DOT1L* itself but increased the expression levels of *POU5F1*, *LIN28*, *SOX2*, *CDX2* and *GATA4* associated with pluripotency and early cell differentiation. In conclusion, DOT1L inhibitor improved early developmental efficiency of porcine SCNT embryos probably via inducing the increased expression of genes important for pluripotency and lineage specification.

## Introduction

Since the first somatic cell cloned animal “Dolly” was born[1], somatic cell nuclear transfer technology (SCNT) has become a forefront focus of life science. Following “Dolly”, SCNT has been successfully established in around 20 different animal species[2]. Pig is a farm animal with significant economic value, and somatic cell cloned pigs were generated more than decade ago [3]. Although pig SCNT has been conducted for over a decade, the cloning efficiency is still less than 5% [4], which is far less than full-term developmental efficiency of naturally fertilized embryos. This rate greatly limits the widespread application of nuclear transfer technology. The low developmental efficiency of SCNT embryos is mainly attributed to the incomplete reprogramming of the donor cell’s nuclear genome during preimplantation embryo development [5].

In natural sexual reproduction, the genome from both parental gametes must undergo profound epigenetic reprogramming to initiate correct gene expression patterns involved in the transition from differentiated gametes into totipotent embryos after fertilization. In the early stages of embryonic development, epigenetic reprogramming events mainly include DNA demethylation, histone modification, chromatin remodeling, genomic imprinting, inactivation of X chromosome and regulation of non-coding RNAs[6]. Furthermore, faithful dynamic changes of epigenetic modifications are also the molecular basis of normal development of SCNT embryos, whereas abnormal epigenetic modifications cause developmental retardation and embryo death. Specifically, abnormalities in DNA methylation and histone acetylation during early development of SCNT embryos have been widely reported [7]. Consistent with these reports, inhibitors of DNA methyltransferases and/or histone deacetylases could improve early and full-term developmental efficiency of SCNT embryos[8,9]. However, whether abnormal histone methylation lead to reduction in reprogramming efficiency needs to be further explored. Recently, methylation of histone H3 at K9 and K79 were reported as reprogramming barriers to the generation of mouse and human induced pluripotent stem cells [10,11]. In mice and humans, reduction in H3K9 trimethylation by over expressing the H3K9 demethylases KDM4A or KDM4B dramatically improved preimplantaion and full-term developmental efficiency of SCNT embryos[12,13]. Additionally, a small molecule inhibitor BIX-01294 that specifically reduces the H3K9me2 levels via repressing histone methyltransferase G9a activity, significantly promoted porcine cloning efficiency[14]. However, whether reduced H3K79 methylation levels also potentially facilitate the early developmental competence and quality of SCNT embryos remains unknown. Therefore, we utilized a highly selective inhibitor (EPZ00477) of a histone H3K79 methyltransferase DOT1L to investigate the function and potential mechanisms of DOT1L during early development of porcine SCNT embryos.

## Materials and methods

All reagents were purchased from Sigma (Sigma-Aldrich, St Louis, MO) unless otherwise specified.

### Ethics statement

The Institutional Animal Care and Use Committee (IACUC) of Anhui Agricultural University specifically approved this study.

### Isolation and culture of fetal fibroblast cells

Donor cells were from an 8 days old male Landrace pig. Ear tissue was harvested, immersed in a small amount of PBS after disinfection, and cut into pieces at approximately 1 mm^3^. The tissue blocks were evenly smeared in a dish and cultured upside down in 37°C, 5% CO_2_ and saturated humidity. After 8 h cell culture medium (15% FBS plus 85% DMEM, 0.1mM NEAA, and 0.05 mM L-glutamine) was added and the samples cultured as mentioned above. Cells were split when 90% confluent. 4th-8th generations of cells were used as donor cells for nuclear transfer.

### *In vitro* oocyte maturation

Ovaries from prepubertal gilts were collected at a local slaughterhouse and transported to the laboratory at 28°C-35°C in physiological saline solution containing penicillin (0.2 IU/mL) and streptomycin sulfate (0.2 IU/mL). The ovaries were washed in saline and the ovarian follicles from 3 to 6 mm in diameter were aspirated using a sterile 10 mL syringe with an 18-gauge needle attached. The aspirated follicular fluid was slowly injected into a preincubated 15 mL centrifuge tube to sediment the cumulus-oocyte complexes (COCs). The COCs with more than three layers of cumulus cells and homogeneous ooplasm were selected under a stereomicroscope. Subsequently, 50 of the COCs were washed and matured in 400 μL *in vitro* maturation (IVM) medium (TCM-199 supplemented with 15% FBS, 10 ng/mL EGF, 10% porcine follicular fluid, 10 IU/mL of eCG, 5 IU/mL of hCG, 0.8 mM L-glutamine and 0.05 mg/mL gentamicin) for 42–44 h at 38.5°C, 5% CO_2_ and saturated humidity. The COCs were treated with DPBS without Ca^2+^and Mg^2+^ (Gibco, Grand Isle, NY) containing 1 mg/mL hyaluronidase to remove the surrounding cumulus cells. Finally, oocytes with clear perivitelline spaces, intact cell membranes, and extruded the first polar body (pb1) were selected for use.

### Somatic cell nuclear transfer

Mature oocytes (containing the first polar body) and a small number of donor cells were placed together in T2 medium (TCM199 plus 2% FBS) containing 7.5 μg / mL cytochalasin B. Oocytes were enucleated with a microinjection needle with an inner diameter of 15–20 μm. Briefly, the microinjection needle was inserted to aspirate the first polar body together with 10%-20% of the adjacent cytoplasm, which should contain the oocyte genomic material. The aspirated cytoplasm was then stained with DAPI to confirm the presence of DNA. If the aspirated cytoplasm had DNA, the remaining cytoplasm should be completely enucleated and could be used to reconstruct the donor cell-cytoplasm pairs. By contrast, if the aspirated cytoplasm did not contain DNA, the remaining cytoplasm should be discarded. Donor cells with a round and slightly burr-like shape were aspirated into the injection needle and one donor cell was injected into the perivitelline space of each enucleated oocyte. The reconstructed oocytes were washed three times in PZM-3, moved to PZM-3 and cultured for 30 min. Then electrofusion was performed on the reconstructed oocytes using electric pulses of 100 μs, 1.56 kv / cm. After electrofusion, the reconstructed oocytes were transferred to PZM-3 and placed in a CO_2_ incubator for 30 min. The reconstructed oocytes were evaluated under a stereomicroscope. The fused embryos were transferred into chemically assisted activation medium (PZM-3 plus 10 μg / mL cycloheximide plus 10 μg / mL cytochalasin B) and incubated at 38.5°C, 5% CO_2_ and saturated humidity for 4 h. Finally, the embryos were transferred to fresh PZM-3 for further culture, the cleavage and blastocyst rates were evaluated after 48 h and 168 h.

### *In vitro* fertilization

IVM medium were equilibrated in a CO_2_ incubator. COC were aspirated and cultured as mentioned earlier. Mature denuded oocytes were transferred to IVF droplets (25 oocytes / 100 μL). Fresh semen stored at 17°C was centrifugally washed with scrubbing solution three times at 225 g × 3 min, and resuspended in *in vitro* fertilization fluid. A volume of semen was transferred to the IVF droplets with COCs securing a sperm concentration of 1.5–5 x10^5^ cell/mL. Sperm and COC were co-incubated for 5 h, washed three times with PZM-3, transferred to a PZM-3 culture dish, and cultured at 38.5°C, 5% CO_2_ and saturated humidity. The cleavage and blastocyst rates were evaluated after 48 h and 168 h.

### DOT1L inhibitor EPZ preparation

Ten mg of EPZ (Selleck Chemicals, S7353) was dissolved in DMSO to create a 50 μM stock solution, and then cryopreserved at -80°C. Then we used porcine embryo culture medium PZM-3 to dilute the EPZ stock solution and prepare the PZM-3 working solutions of 50, 5 and 0.5 nM EPZ. Furthermore, 1 μL DMSO was added into 1 mL PZM-3 as the negative control.

### Immunofluorescence staining

Embryos were fixed in 4% paraformaldehyde (PFA) for 15 min, and then washed three times with DPBS. Zona pellucida was removed with 2N hydrochloric acid, followed by washing three times with DPBS. They were then placed in 1% Triton-X100 for permeabilization. The embryos were washed three times with DPBS. After permeabilization, the embryos were incubated in 2% BSA and blocked for 1h. Then they were incubated at room temperature for 1 h with the primary antibody to detect H3K79me2 (Abcam, ab3594) at 1: 200. The embryos were washed three times with DPBS. Then they were incubated at room temperature for 1 h with the secondary antibody Alexa Fluor-488 (Invitrogen, A11008) at 1: 200. The embryos were washed three times with DPBS. DNA counterstaining was done with 10 μl /mL of propidium iodide (Sigma, P4170) for 10 min. Finally, the embryos were washed 3 times with DPBS and mounted on glass slides with a small drop of Vectashield (Vector-Lab, H-1000) mounting medium, and observed under a confocal laser-scanning microscope (Olympus, FluoView1000). All operations were carried out at room temperature, and Image J (1.49v published by NIH of USA in 2015) was used to calculate the fluorescence intensity.

### RNA extraction and reverse transcription

For each biological replicates, total RNA was isolated from the pooled 10 SCNT blastocysts (n = 10) using RNeasy Micro Kit (Qiagen, 74004). The extracted RNA was quantified with spectrophotometry at 260/280 nm with a NanoDrop 2000 instrument (Thermo Scientific, Waltham, MA, USA). Reverse transcription was immediately performed using QuantiTect Reverse Transcription Kit (Qiagen, 205311). The complementary DNA was aliquoted and stored at -80°C, until ready for use. The samples were collected three times and three biological replicates were conducted for each gene.

### Quantitative real-time PCR

The primer sequences used were listed in S1 Table. Polymerase chain reactions (PCRs) were prepared using FastStart SYBR Green Master (Roche, 04673514001) and performed using StepOne Plus (Applied Biosystems). Each reaction consisted of 1.5 μl complementary DNA, 7.5 μL 2 × SYBR Green PCR master mix, 0.9 μl of each primer pair, and 5.1 μl ultrapure water. The housekeeping gene *EF1A1* was used as the reference gene. The amplification conditions were as follows: pre-denaturation at 95°C for 10 min, followed by 45 amplification cycles of 95°C denaturation for 15 seconds, 60°C annealing for 10 seconds, and 72°C extension for 20 seconds. Three technical replicates were conducted in each PCR reaction.

### Statistical analysis

All experiments were repeated at least three times. The data were presented as mean ± standard error (mean ± S.E.M) and SPSS (Version 17.0) was used to do single factor analysis of variance (ANOVA) of the cleavage rate, blastocyst rate and total cell number per blastocyst and others, *p<*0.05 was considered as a significant difference.

## Results

### DOT1L inhibitor EPZ improves early developmental efficiency of porcine SCNT embryos

**Fig 1:**
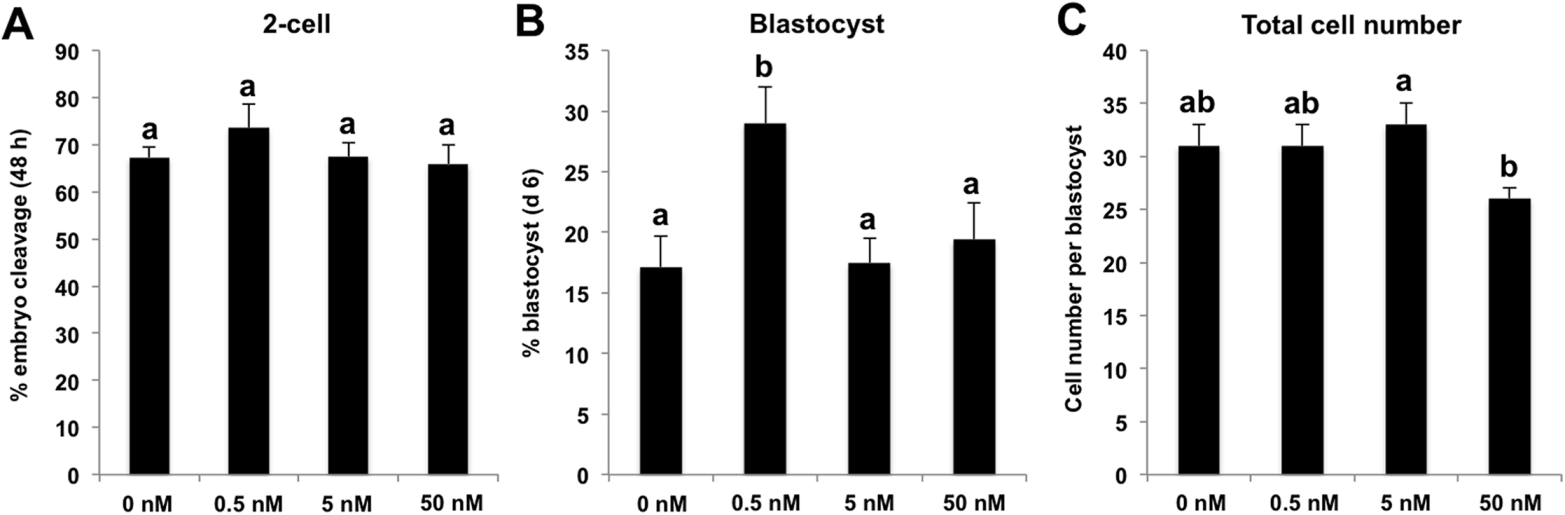
Effect of EPZ treatment with different dosages for 24 h on developmental competence of porcine SCNT embryos. Porcine SCNT pronuclear stage embryos were cultured in PZM-3 medium supplemented with EPZ at different concentrations, namely 0 (n = 96), 0.5 (n = 96), 5 (n = 95) and 50 nM (n = 95). The number of 2-cell (48 h after activation, n = 64, 70, 64 and 63) **(A)**, blastocysts (day 6 after activation, n = 17, 28, 17 and 19) **(B)** and total cell number per blastocyst (n = 17, 28, 17 and 19) **(C)** were recorded and statistically analyzed by SPSS software. The experiment was repeated four times for each group. Data are expressed as mean ± S.E.M. Values with different superscripts across treatments indicate significant differences (*p <* 0.05).

Although DOT1L was identified as a negative regulator of somatic cell reprogramming during the generation of induced pluripotent stem cells [11], the role of DOT1L during nuclear transfer-mediated cellular reprogramming is still unknown. To determine whether DOT1L inhibition using a selective inhibitor (referred to here as EPZ) improves early developmental efficiency and quality of porcine somatic cell cloned embryos, we cultured SCNT pronuclear stage embryos with EPZ at different concentrations and durations. First, we compared the developmental rates and quality of SCNT embryos cultured in PZM-3 medium supplemented with EPZ of different dosages (0, 0.5, 5 and 50 nM) for 24 h. We found that the cleavage rate (48 h after activation) and total cell number per blastocyst from the three treatment groups (0.5, 5 and 50 nM) were similar to that in the control group (Fig 1A and 1C). Interestingly, total cell number per blastocyst in the 50 nM group was significantly lower than that in the 5 nM group (Fig 1C). Consistent with the result, we found that high dosage groups (5 and 50 nM) did not improve the blastocyst rate (day 6 after activation), and we only observed that 0.5 nM treatment increased the blastocyst rate compared to other treatment and control groups (*p <* 0.05) (Fig 1B).

**Fig 2:**
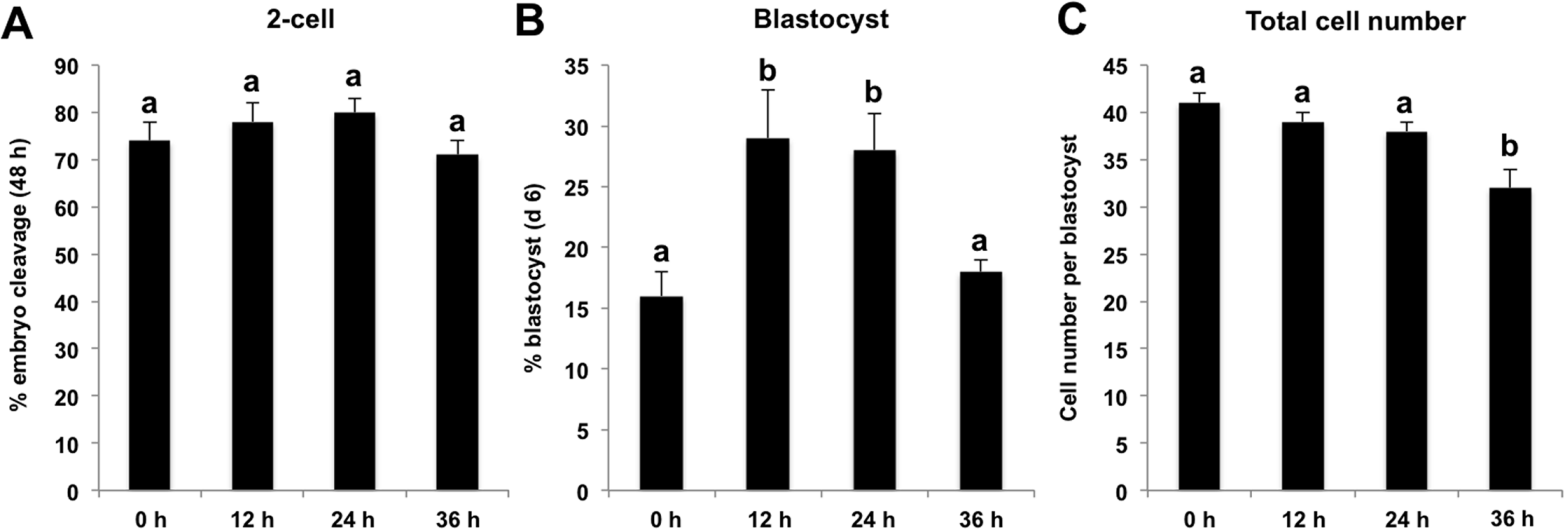
Effect of 0.5 nM EPZ treatment with different durations on developmental efficiency of porcine SCNT embryos. Porcine SCNT pronuclear stage embryos were cultured in PZM-3 medium supplemented with 0.5 nM EPZ for different durations, namely 0 (n = 97), 12 (n = 97), 24 (n = 88) and 36 h (n = 100). The number of 2-cell (48 h after activation, n = 72, 76, 70 and 70) **(A)**, blastocysts (day 6 after activation, n = 16, 27, 25 and 18) **(B)** and total cell number per blastocyst (n = 16, 27, 25 and 18) **(C)** were recorded and statistically analyzed by SPSS software. The experiment was repeated four times for each group. Data are expressed as mean ± S.E.M. Values with different superscripts across treatments indicate significant differences (*p <* 0.05).

Next, we wanted to determine whether 0.5 nM EPZ with different treatment durations affects early developmental competence and quality of porcine SCNT embryos. We cultured SCNT pronuclear stage embryos in PZM-3 medium supplemented with 0.5 nM EPZ for 0, 12, 24 or 36 h. We did not observe a significant difference for the cleavage rate (Fig 2A). However, the blastocyst rates in the 12 and 24 h treatment groups were significantly higher than that of control group and of 36 h treatment group (*p <* 0.05) (Fig 2B). Surprisingly, total cell number per blastocyst in the 36 h treatment group was significantly lower than that of the other three groups (*p <* 0.05) (Fig 2C), but that in 12 and 24 h treatment groups did not differ from the control group. Taken together, these data indicate that EPZ with optimal dosage and duration can improve early developmental efficiency of porcine SCNT embryos.

### DOT1L inhibitor EPZ reduces H3K79me2 level in porcine SCNT pronuclear stage embryos

**Fig 3:**
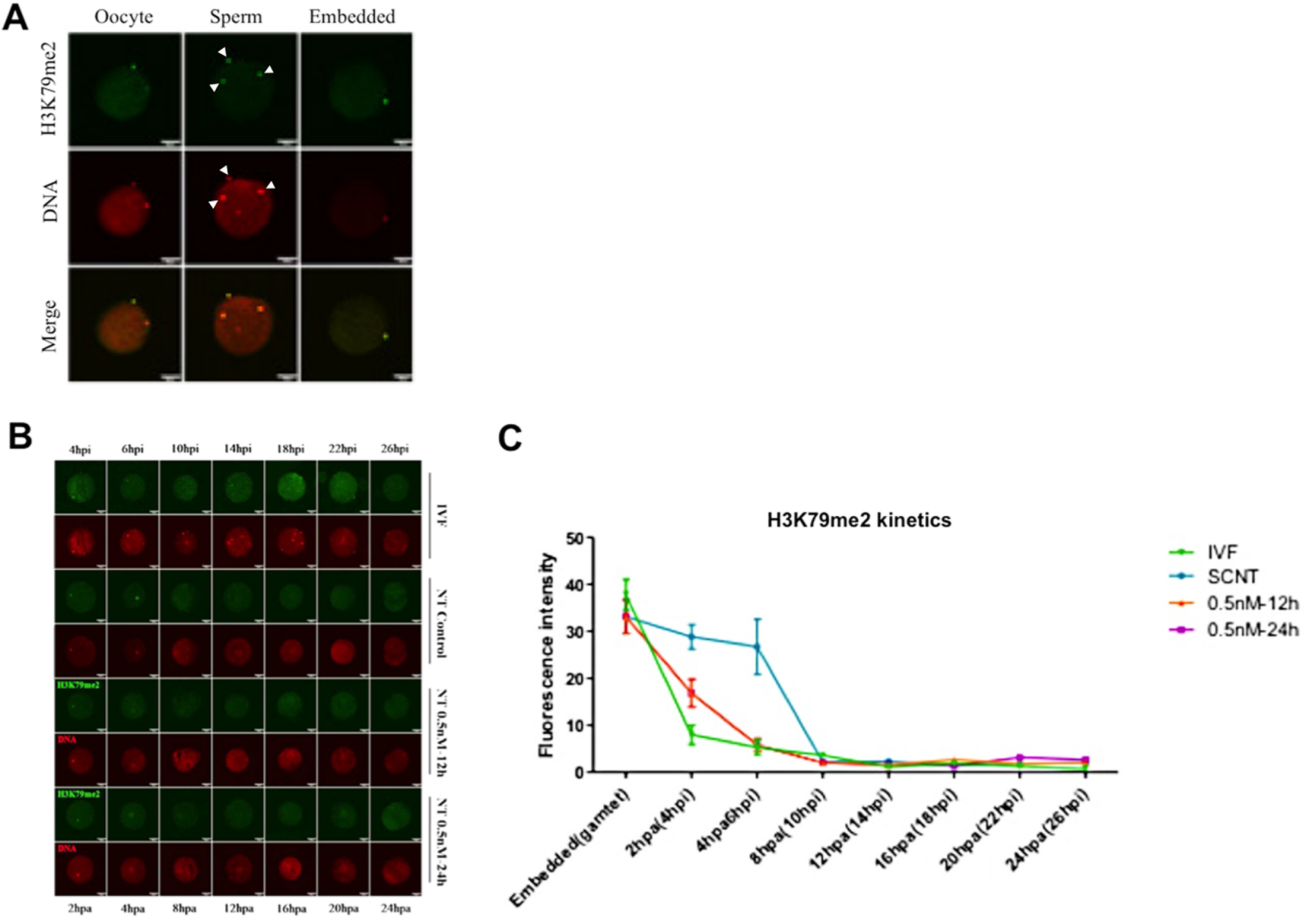
DOT1L inhibition reduces H3K79me2 level in porcine SCNT pronuclear embryos. **(A)** Representative images of H3K79me2 in porcine MII oocytes, sperms and cytoplasm-donor cell reconstructed couplets immunostained with anti-H3K79me2 antibody. Primary antibody was localized with Alexa Flour 488-conjugated secondary antibody (green). DNA was stained with propidium iodide (red). Bottom panels showed the merged images (yellow) between H3K79me2 signals (green) and DNA staining (red). White arrowhead denotes localization of H3K79me2 and DNA in sperm. Scale bars = 50μm. **(B)** Representative images of porcine IVF and SCNT pronuclear embryos at different developmental stages. IVF embryos were fixed from 4 hpi to 26 hpi simultaneously corresponding to different stages from 2 hpa to 24 hpa in SCNT embryos. All embryos were immunostained with anti-H3K79me2 antibody. Primary antibody was localized with Alexa Flour 488-conjugated secondary antibody (green). DNA was stained with propidium iodide (red). Scale bars = 50μm. **(C)** Quantification of H3K79me2 fluorescence intensity between IVF and SCNT pronuclear embryos from A. Green line denotes IVF group, blue line represents SCNT control group, yellow line indicates SCNT embryos treated with 0.5 nM EPZ for 12 h and purple line marks SCNT embryos treated with 0.5 nM EPZ for 24 h. Data are expressed as mean ± S.E.M.

To determine whether DOT1L activity inhibition reduces H3K79me2 level in porcine early SCNT embryos, we performed immunofluorescence staining using an antibody that specifically recognizes the H3K79me2 antigen in metaphase II oocytes and early embryos. Specificity of the commercially available H3K79me2 antibody was confirmed by a pre-absorption test using the antigen peptide in *in vitro* fertilization blastocysts (S1 Fig). The data revealed that the H3K79me2 mark is highly enriched in MII oocytes (Fig 3A). At the same time, we quantitatively analyzed the dynamic changes of H3K79me2 in the pronuclear stage embryos derived from IVF, SCNT-negative control and SCNT embryos-treated with 0.5 nM EPZ for 12 h or 24 h (Fig 3B and 3C). We found that H3K79me2 levels in SCNT early embryos after activation was higher (*p <* 0.05) than that in IVF counterparts (Fig 3B and 3C). However, H3K79me2 level in SCNT pronuclear embryos treated with 0.5 nM EPZ for 12 h or 24 h was decreased and comparable to IVF embryos from 2 h to 10 h post-insemination (Fig 3C). These results demonstrate that inhibition of DOT1L activity using 0.5 nM EPZ for 12 h or 24 h can reduce the H3K79me2 level in SCNT embryos at the pronuclear stage.

### DOT1L inhibitor EPZ increases the expression levels of genes important for pluripotency establishment and lineage specification

**Fig 4:**
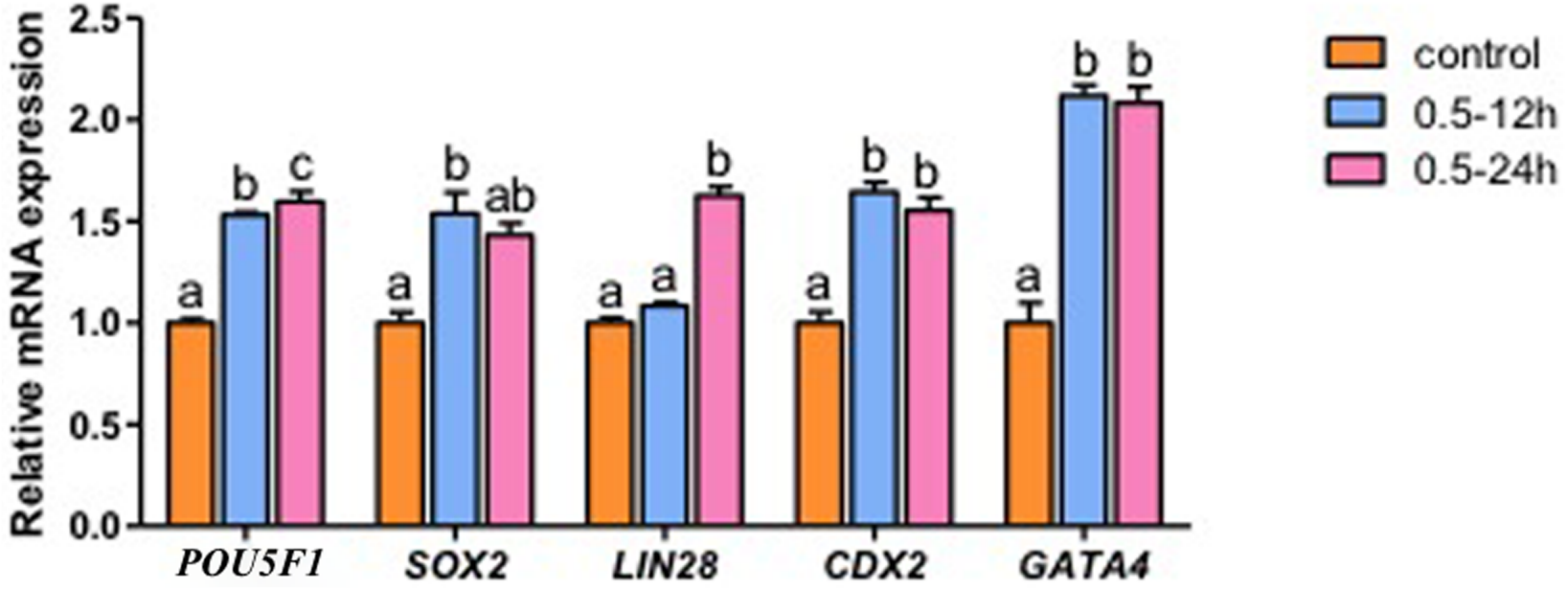
DOT1L inhibition enhances expression levels of genes important for pluripotency establishment and lineage specification. Relative mRNA levels of *POU5F1*, *LIN28*, *SOX2*, *CDX2* and *GATA4* in SCNT blastocysts. Expression levels were determined by quantitative RT-PCR and were normalized against endogenous housekeeping gene *EF1α1*. Orange bars indicate SCNT control group, blue bars denote SCNT embryos treated with 0.5 nM EPZ for 12 h and pink bars represent SCNT embryos treated with 0.5 nM EPZ for 24 h. Data are shown as mean ± S.E.M. Values with different superscripts across groups indicate significant differences (*p <* 0.05).

Given the observed beneficial effect of 0.5 nM EPZ supplement for 12 h or 24 h on early development of porcine SCNT embryos, we speculated that DOT1L inhibition could affect the expression levels of genes associated with pluripotency establishment and lineage specification underlying early embryonic development. To test this hypothesis, we utilized qRT-PCR to evaluate the expression of *POU5F1* and *LIN28* involved in establishing embryonic pluripotency. The abundance of *POU5F1* transcripts was significantly increased in SCNT blastocysts treated with 0.5 nM EPZ for 12 and 24 h (*p <* 0.05) (Fig 4) and the levels of *LIN28* transcripts were also increased in SCNT blastocysts treated with 0.5 nM EPZ for 24 h, suggesting that endogenous DOT1L is a negative regulator of *POU5F1* and *LIN28* during early development of porcine SCNT embryos. At the same time, to rule out the possibility that the DOT1L inhibitor could alter the expression of endogenous *DOT1L* in SCNT embryos, qRT-PCR analysis demonstrated that the levels of *DOT1L* mRNA were unchanged in EPZ-treated SCNT blastocysts (S2 Fig). To further investigate the molecular mechanisms underlying the increase in developmental competence of cloned embryos after EPZ treatment, we examined the expression of genes necessary for the specification of the inner cell mass (*SOX2*), trophectoderm (*CDX2*) and primitive endoderm (*GATA4*) lineages in mammalian blastocysts. We observed a significant upregulation in the expression levels of *SOX2*, *CDX2* and *GATA4* in EPZ-treated SCNT blastocysts (*p <* 0.05) (Fig 4). Altogether, these results suggest that inhibition of DOT1L activity using EPZ can induce the increased expression of genes important for pluripotency and lineage specification during early development of porcine SCNT embryos.

## Discussion

Although inhibition of DOT1L activity using the selective pharmacological inhibitor EPZ could improve the production efficiency and quality of induced pluripotent stem cells in mice and humans [11], the function of DOT1L in nuclear transfer-mediated somatic cell reprogramming was not yet determined. In the present study, we found that the inhibitor of the H3K79 methyltransferase DOT1L could significantly enhance early developmental efficiency of porcine SCNT embryos. However, EPZ treatment did not improve the total cell number of porcine SCNT blastocysts. The reason for this discrepancy could be that a low concentration of EPZ in a short developmental time window did not accelerate the cell cycle during early development of SCNT embryos. On the other hand, EPZ at a high concentration impaired early embryonic development due to the toxicity effect of this drug itself. Consistent with this result, previous research demonstrated that 5-aza-dC [15], BIX-01294 [16] and TSA [17] also had negative effects on embryonic development.

Epigenetic reprogramming is necessary for normal development of embryos after fertilization and this dynamic reprogramming mainly occurs during early embryonic development. Therefore, SCNT embryos have to precisely recapitulate the dynamic reprograming of many epigenetic modifications attached on the chromatin of donor cells to ensure the normal development of SCNT embryos. However, abnormal epigenetic modifications leading to developmental failure of cloned embryos were often observed[9,14,18]. For example, DNA and histone methylation levels in bovine and ovine cloned embryos were higher than that in IVF counterparts[7,19]. Interestingly, maternally provided DOT1L protein quickly decreased at the onset of fertilization and even was completely absent in 2-cell embryos[20]. Concurrently, H3K79me2 signals disappear soon after fertilization in mice [21]. In line with this phenomenon, we also observed that H3K79me2 signals were undetectable soon after fertilization in pigs. Previous studies indicated that the rapid absence of DOT1L protein and H3K79me2, which is involved in the rearrangement of major satellite repeat and nucleolar precursor bodies (NPBs) to form the specific heterochromatin configuration, is necessary for early development of naturally fertilized and SCNT embryos [22,23]. Surprisingly, we found that H3K79me2 level in porcine SCNT 1-cell embryos was higher than that in IVF-derived embryos. Perhaps the abnormal high expression of H3K79me2 could cause the dissociation of major satellite repeat and NPBs, which lead to the aberrant expression patterns of genes important for early embryonic development. As we expected, the DOT1L inhibitor EPZ significantly reduced the level of H3K79me2 in SCNT 1-cell embryos to a similar level as that in IVF counterparts and dramatically enhanced the developmental efficiency of porcine SCNT embryos. However, EPZ only suppressed the activity of DOT1L protein rather than affecting its expression at the RNA level.

A previous study reported that DOT1L-mediated H3K79 methylation could act as a transcriptional repressor of core pluripotent genes in mice [11]. Consistent with this report, qRT-PCR analysis demonstrated that DOT1L inhibitor treatment significantly elevated the expression levels of *POU5F1*, *LIN28*, *SOX2*, *CDX2* and *GATA4*. In mouse preimplantation development, depletion of zygotic *POU5F1* impaired the proliferation and specification of ICM lineage in blastocysts outgrowth assay [24]. At the same time, *POU5F1* and *LIN28* are required for efficient reprogramming of fibroblast cells to induced pluripotent stem cells [25], indicating that POU5F1 and LIN28 can promote the pluripotency establishment. Furthermore, previous studies indicated SOX2 is the earliest and most reliable molecular marker for ICM lineage in mice [26] and pigs [27]. Even disruption of *SOX2* function via RNAi leads to the failure of mouse blastocyst formation due to defective trophectoderm lineage [28], suggesting that SOX2 may play a critical role during early embryonic development. Likewise, ablation of zygotic *CDX2* or *GATA4* resulted in the failure of functional trophectoderm and primitive endoderm development which indirectly prevented expanding progression of blastocysts [29,30], suggesting that transcription factor CDX2 and GATA4 are master regulators for specification of trophectoderm and primitive endoderm in early embryos. On the basis of these previous data, the increased expression of *POU5F1*, *LIN28*, *SOX2*, *CDX2* and *GATA4* gene transcripts might contribute to the improvements in developmental competence of porcine SCNT embryos due to somatic cell-inherited H3K79 demethylation after EPZ treatment.

Taken together, our data indicate that EPZ treatment enhanced early developmental competence of porcine SCNT embryos potentially through improvements in epigenetic reprogramming of H3K79me2 and transcriptional expression of genes important for pluripotency establishment and lineage specification. Because various epigenetic defects occurred during somatic cell reprogramming, further studies are required to identify which cluster of genes or epigenetic loci are dysregulated in somatic cell reprogramming to further improve the cloning efficiency in pigs.

## Supporting information

S1 Fig Verification of H3K79me2 antibody specificity. The commercial H3K79me2 primary antibody was preincubated with (+) or without (−) antigen peptide (Abcam, catalog no. ab4556, v/v = 5:1) at room temperature for 1.5 h before the incubation with IVF blastocysts. H3K79me2 signals were observed in blastocysts using unabsorbed primary antibody. By contrast, H3K79me2 signals were absent in blastocysts using pre-absorbed primary antibody. H3K79me2 antibody was localized with Alexa Flour 488-conjugated secondary antibody (green). DNA was stained with propidium iodide (red). Bottom panels showed the merged images (yellow) between H3K79me2 signals (green) and DNA staining (red). Scale bars = 50μm.
(DOCX)

S2 Fig DOT1L inhibition does not alter the expression levels of *DOT1L*. qRT-PCR analysis of *DOT1L* in SCNT blastocysts. Expression levels were normalized against endogenous housekeeping gene *EF1α1*. Orange bar indicates SCNT control blastocysts without EPZ treatment and blue bar denotes SCNT blastocysts treated with 0.5 nM EPZ for 24 h. Data are shown as mean ± S.E.M.
(DOCX)

S1 Table Sequence information on porcine-specific primers for quantitative real-time polymerase chain reaction. (DOCX)

## References

[1] Wilmut I, Schnieke AE, McWhir J, Kind AJ, Campbell KH. Viable offspring derived from fetal and adult mammalian cells. Nature. 1997;385(6619): 810–3. doi: 10.1038/385810a0 9039911 10.1038/385810a0

[2] Loi P, Iuso D, Czernik M, Ogura A. A New, Dynamic Era for Somatic Cell Nuclear Transfer? Trends Biotechnol. 2016;34(10): 791–7. doi: 10.1016/j.tibtech.2016.03.008 27118511 10.1016/j.tibtech.2016.03.008

[3] Yin XJ, Tani T, Yonemura I, Kawakami M, Miyamoto K, Hasegawa R, et al Production of cloned pigs from adult somatic cells by chemically assisted removal of maternal chromosomes. Biol Reprod. 2002;67(2): 442–6. 12135879 10.1095/biolreprod67.2.442

[4] Jin JX, Li S, Gao QS, Hong Y, Jin L, Zhu HY, et al Significant improvement of pig cloning efficiency by treatment with LBH589 after somatic cell nuclear transfer. Theriogenology. 2013;80(6): 630–5. doi: 10.1016/j.theriogenology.2013.06.006 23866857 10.1016/j.theriogenology.2013.06.006

[5] Sepulveda-Rincon LP, Solanas Edel L, Serrano-Revuelta E, Ruddick L, Maalouf WE, Beaujean N. Early epigenetic reprogramming in fertilized, cloned, and parthenogenetic embryos. Theriogenology. 2016;86(1): 91–8. doi: 10.1016/j.theriogenology.2016.04.022 27156679 10.1016/j.theriogenology.2016.04.022

[6] Li E. Chromatin modification and epigenetic reprogramming in mammalian development. Nat Rev Genet. 2002;3(9): 662–73. doi: 10.1038/nrg887 12209141 10.1038/nrg887

[7] Kang YK, Koo DB, Park JS, Choi YH, Chung AS, Lee KK, et al Aberrant methylation of donor genome in cloned bovine embryos. Nat Genet. 2001;28(2): 173–7. doi: 10.1038/88903 11381267 10.1038/88903

[8] Huan YJ, Zhu J, Xie BT, Wang JY, Liu SC, Zhou Y, et al Treating cloned embryos, but not donor cells, with 5-aza-2'-deoxycytidine enhances the developmental competence of porcine cloned embryos. J Reprod Dev. 2013;59(5): 442–9. doi: 10.1262/jrd.2013-026 23748715 10.1262/jrd.2013-026 PMC3934119

[9] Zhao J, Hao Y, Ross JW, Spate LD, Walters EM, Samuel MS, et al Histone deacetylase inhibitors improve in vitro and in vivo developmental competence of somatic cell nuclear transfer porcine embryos. Cell Reprogram. 2010;12(1): 75–83. doi: 10.1089/cell.2009.0038 20132015 10.1089/cell.2009.0038 PMC2842950

[10] Chen J, Liu H, Liu J, Qi J, Wei B, Yang J, et al H3K9 methylation is a barrier during somatic cell reprogramming into iPSCs. Nat Genet. 2013;45(1): 34–42. doi: 10.1038/ng.2491 23202127 10.1038/ng.2491

[11] Onder TT, Kara N, Cherry A, Sinha AU, Zhu N, Bernt KM, et al Chromatin-modifying enzymes as modulators of reprogramming. Nature. 2012;483(7391): 598–602. doi: 10.1038/nature10953 22388813 10.1038/nature10953 PMC3501145

[12] Matoba S, Liu Y, Lu F, Iwabuchi KA, Shen L, Inoue A, et al Embryonic development following somatic cell nuclear transfer impeded by persisting histone methylation. Cell. 2014;159(4): 884–95. doi: 10.1016/j.cell.2014.09.055 25417163 10.1016/j.cell.2014.09.055 PMC4243038

[13] Chung YG, Matoba S, Liu Y, Eum JH, Lu F, Jiang W, et al Histone Demethylase Expression Enhances Human Somatic Cell Nuclear Transfer Efficiency and Promotes Derivation of Pluripotent Stem Cells. Cell Stem Cell. 2015;17(6): 758–66. doi: 10.1016/j.stem.2015.10.001 26526725 10.1016/j.stem.2015.10.001

[14] Huang J, Zhang H, Yao J, Qin G, Wang F, Wang X, et al BIX-01294 increases pig cloning efficiency by improving epigenetic reprogramming of somatic cell nuclei. Reproduction. 2016;151(1): 39–49. doi: 10.1530/REP-15-0460 26604326 10.1530/REP-15-0460

[15] Enright BP, Kubota C, Yang X, Tian XC. Epigenetic characteristics and development of embryos cloned from donor cells treated by trichostatin A or 5-aza-2'-deoxycytidine. Biol Reprod. 2003;69(3): 896–901. doi: 10.1095/biolreprod.103.017954 12748129 10.1095/biolreprod.103.017954

[16] Fu L, Zhang J, Yan FX, Guan H, An XR, Hou J. Abnormal histone H3K9 dimethylation but normal dimethyltransferase EHMT2 expression in cloned sheep embryos. Theriogenology. 2012;78(9): 1929–38. doi: 10.1016/j.theriogenology.2012.07.017 23058792 10.1016/j.theriogenology.2012.07.017

[17] Kishigami S, Mizutani E, Ohta H, Hikichi T, Thuan NV, Wakayama S, et al Significant improvement of mouse cloning technique by treatment with trichostatin A after somatic nuclear transfer. Biochem Biophys Res Commun. 2006;340(1): 183–9. doi: 10.1016/j.bbrc.2005.11.164 16356478 10.1016/j.bbrc.2005.11.164

[18] Jin L, Guo Q, Zhu HY, Xing XX, Zhang GL, Xuan MF, et al Quisinostat treatment improves histone acetylation and developmental competence of porcine somatic cell nuclear transfer embryos. Mol Reprod Dev. 2017. 10.1002/mrd.22787 28224725

[19] Dean W, Santos F, Stojkovic M, Zakhartchenko V, Walter J, Wolf E, et al Conservation of methylation reprogramming in mammalian development: aberrant reprogramming in cloned embryos. Proc Natl Acad Sci U S A. 2001;98(24): 13734–8. doi: 10.1073/pnas.241522698 11717434 10.1073/pnas.241522698 PMC61110

[20] Ooga M, Suzuki MG, Aoki F. Involvement of DOT1L in the remodeling of heterochromatin configuration during early preimplantation development in mice. Biol Reprod. 2013;89(6): 145 doi: 10.1095/biolreprod.113.113258 24132959 10.1095/biolreprod.113.113258

[21] Ooga M, Inoue A, Kageyama S, Akiyama T, Nagata M, Aoki F. Changes in H3K79 methylation during preimplantation development in mice. Biol Reprod. 2008;78(3): 413–24. doi: 10.1095/biolreprod.107.063453 18003948 10.1095/biolreprod.107.063453

[22] Akiyama T, Suzuki O, Matsuda J, Aoki F. Dynamic replacement of histone H3 variants reprograms epigenetic marks in early mouse embryos. PLoS Genet. 2011;7(10): e1002279 doi: 10.1371/journal.pgen.1002279 21998593 10.1371/journal.pgen.1002279 PMC3188537

[23] Maalouf WE, Liu Z, Brochard V, Renard JP, Debey P, Beaujean N, et al Trichostatin A treatment of cloned mouse embryos improves constitutive heterochromatin remodeling as well as developmental potential to term. BMC Dev Biol. 2009;9(11. 10.1186/1471-213X-9-11 PMC2656487 19210795

[24] Nichols J, Zevnik B, Anastassiadis K, Niwa H, Klewe-Nebenius D, Chambers I, et al Formation of pluripotent stem cells in the mammalian embryo depends on the POU transcription factor POU5F1. Cell. 1998;95(3): 379–91. 9814708 10.1016/s0092-8674(00)81769-9

[25] Yu J, Vodyanik MA, Smuga-Otto K, Antosiewicz-Bourget J, Frane JL, Tian S, et al Induced pluripotent stem cell lines derived from human somatic cells. Science. 2007;318(5858): 1917–20. doi: 10.1126/science.1151526 18029452 10.1126/science.1151526

[26] Guo G, Huss M, Tong GQ, Wang C, Li Sun L, Clarke ND, et al Resolution of cell fate decisions revealed by single-cell gene expression analysis from zygote to blastocyst. Dev Cell. 2010;18(4): 675–85. doi: 10.1016/j.devcel.2010.02.012 20412781 10.1016/j.devcel.2010.02.012

[27] Liu S, Bou G, Sun R, Guo S, Xue B, Wei R, et al Sox2 is the faithful marker for pluripotency in pig: evidence from embryonic studies. Dev Dyn. 2015;244(4): 619–27. doi: 10.1002/dvdy.24248 25619399 10.1002/dvdy.24248

[28] Keramari M, Razavi J, Ingman KA, Patsch C, Edenhofer F, Ward CM, et al Sox2 is essential for formation of trophectoderm in the preimplantation embryo. PLoS One. 2010;5(11): e13952 doi: 10.1371/journal.pone.0013952 21103067 10.1371/journal.pone.0013952 PMC2980489

[29] Strumpf D, Mao CA, Yamanaka Y, Ralston A, Chawengsaksophak K, Beck F, et al Cdx2 is required for correct cell fate specification and differentiation of trophectoderm in the mouse blastocyst. Development. 2005;132(9): 2093–102. doi: 10.1242/dev.01801 15788452 10.1242/dev.01801

[30] Soudais C, Bielinska M, Heikinheimo M, MacArthur CA, Narita N, Saffitz JE, et al Targeted mutagenesis of the transcription factor GATA-4 gene in mouse embryonic stem cells disrupts visceral endoderm differentiation in vitro. Development. 1995;121(11): 3877–88. 8582296 10.1242/dev.121.11.3877

