# Supplementary figures and images for: DOT1L inhibitor improves early development of porcine somatic cell nuclear transfer embryos
Source: PLoS One. 2017 Jun 20;12(6):e0179436. doi: 10.1371/journal.pone.0179436 (PMC5478106; doi:10.1371/journal.pone.0179436)

**S1_Fig.**


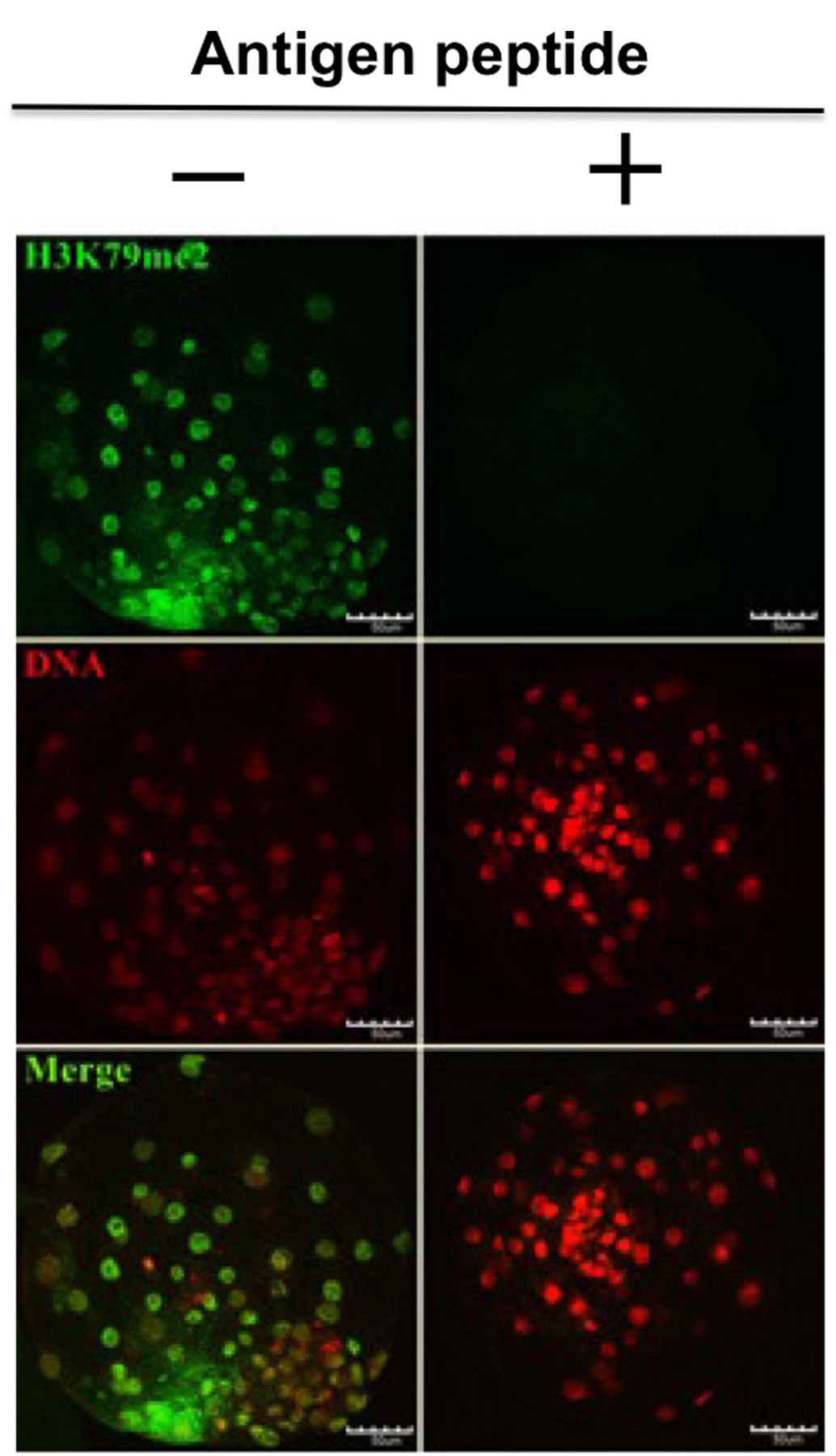

Supplement: S1 Fig — The commercial H3K79me2 primary antibody was preincubated with (+) or without (−) antigen peptide (Abcam, catalog no. ab4556, v/v = 5:1) at room temperature for 1.5 h before the incubation with IVF blastocysts. H3K79me2 signals were observed in blastocysts using unabsorbed primary antibody. By contrast, H3K79me2 signals were absent in blastocysts using pre-absorbed primary antibody. H3K79me2 antibody was localized with Alexa Flour 488-conjugated secondary antibody (green). DNA was stained with propidium iodide (red). Bottom panels showed the merged images (yellow) between H3K79me2 signals (green) and DNA staining (red). Scale bars = 50μm. (DOCX) [file pone.0179436.s001.docx]

**S2_Fig.**
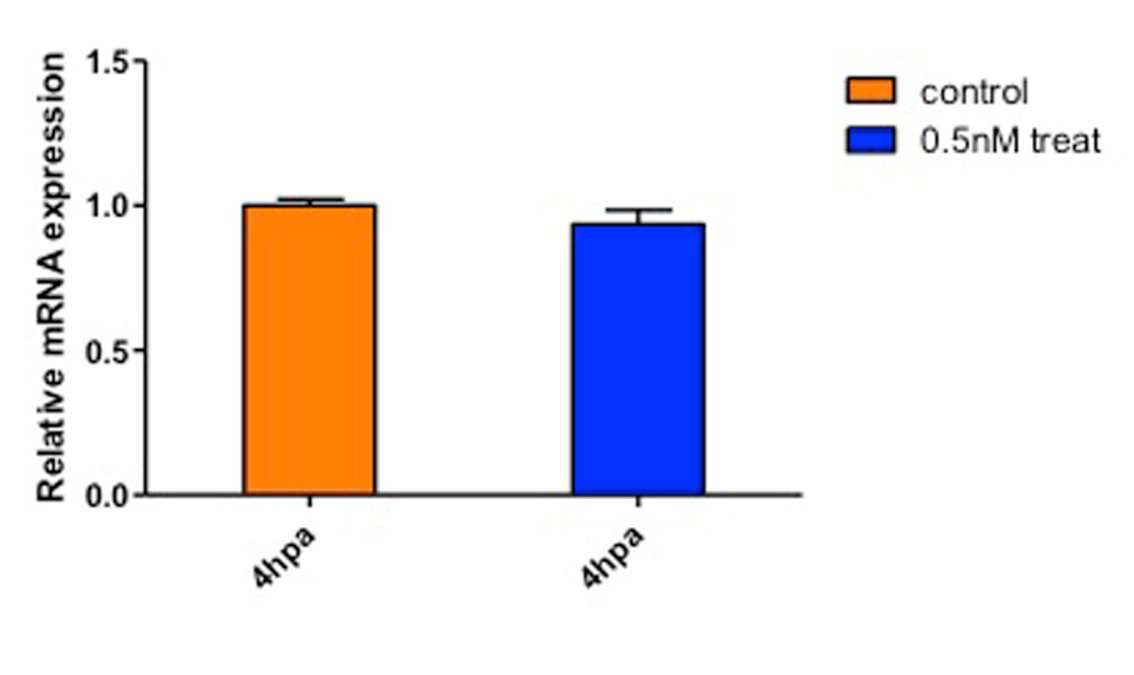

Supplement: S2 Fig — qRT-PCR analysis of DOT1L in SCNT blastocysts. Expression levels were normalized against endogenous housekeeping gene EF1α1. Orange bar indicates SCNT control blastocysts without EPZ treatment and blue bar denotes SCNT blastocysts treated with 0.5 nM EPZ for 24 h. Data are shown as mean ± S.E.M. (DOCX) [file pone.0179436.s002.docx]
