# Supplementary material for: DOT1L inhibitor improves early development of porcine somatic cell nuclear transfer embryos
Source: PLoS One. 2017 Jun 20;12(6):e0179436. doi: 10.1371/journal.pone.0179436 (PMC5478106; doi:10.1371/journal.pone.0179436)
Supplement: S1 Table — (DOCX) [file pone.0179436.s003.docx]

**S1 Table.** **Sequence information on porcine-specific primers for quantitative real-time polymerase chain reaction.**

| Gene | Primer sequence (5ˊ-3ˊ) | Annealing temp (℃) | GenBank accession no. |
| --- | --- | --- | --- |
| p*DOT1L* | F: TGTCGCTGGCCTCTTCACT  R: GGGATGAGATGGCCGTGAT | 60 | CV876698.1 |
| p*POU5F1* | F: CAAACTGAGGTGCCTGCCCTTC  R: ATTGAACTTCACCTTCCCTCCAACC | 60 | NM_001113060.1 |
| p*SOX2* | F: CCCGTGGTTACCTCTTCTTCC  R: TACCGTTGATGGCCGTGCC | 60 | NM_001123197.1 |
| p*LIN28* | F: CAGAGTAAGCTGCACATGGAGG  R: GTAGGCTGGCTTTCCCTGTG | 60 | EU503118.1 |
| p*CDX2* | F: CAGGACAGGGCCTTGTTTAG  R: CAGGTTGGCTCTGGCATTT | 60 | XM_003130908.2 |
| p*GATA4* | F: AAACGGAAGCCCAAGAACCT  R: GGCCAGACATGGCACTAACTG | 60 | NM_214293.1 |
| *EF1α1* | F: AATGCGGTGGGATCGACAAA  R: CACGCTCACGTTCAGCCTTT | 60 | NM_001097418.1 |

Abbreviations: F, forward; R, reverse.
